# 5‐Hydroxymethylcytosine profiling from genomic and cell‐free DNA for colorectal cancers patients

**DOI:** 10.1111/jcmm.14252

**Published:** 2019-03-26

**Authors:** Pingting Gao, Shengli Lin, Mingyan Cai, Yan Zhu, Yanqun Song, Yi Sui, Jing Lin, Jiaxiuyu Liu, Xingyu Lu, Yunshi Zhong, Yuehong Cui, Pinghong Zhou

**Affiliations:** ^1^ Zhongshan Hospital, Endoscopy Center and Endoscopy Research Institute, Fudan University Shanghai China; ^2^ Shanghai Epican Genetech Co. Ltd., Zhangjiang Hi‐Tech Park Shanghai China; ^3^ Shanghai Epican Biotech Co.Ltd. Shanghai China; ^4^ Medical Oncology Department Zhongshan Hospital, Fudan University Shanghai China

**Keywords:** 5‐hydroxymethylcytosine, colon cancer, hmC‐Seal, precancerous adenoma

## Abstract

5‐Hydroxymethylcytosine (5hmC) is a DNA modification that is generated by the oxidation of 5‐methylcytosine (5mC) in a reaction catalyzed by the ten‐eleven translocation (TET) family enzymes. It tends to mark gene activation and affects a spectrum of developmental and disease‐related biological processes. In this manuscript, we present a 5hmC selective chemical labelling technology (hmC‐Seal) to capture and sequence 5hmC‐containing DNA fragments with low input. We tested 10 tumour/adjacent colon cancer tissues and 10 tumour/healthy plasma samples. Furthermore, we tested if this methodology could generate the 5hmC differential genes among cancer patients, healthy controls and precancerous adenoma patients from plasma. Robust cancer‐specific epigenetic signatures were identified for colon cancers. The results show that 5hmC is mainly distributed in gene active regions. The results also indicate the potential application of 5hmC change signals in early stage of colon cancer, even show potential in the diagnosis of precancerous adenoma. We demonstrated the robustness of the 5hmC‐Seal method in tissue and cell‐free DNA (cfDNA) as potential biomarkers. Moreover, this study provides the potential value and feasibility of 5hmC‐Seal approach on colorectal cancer (CRC) early detection. We believe this strategy could be an effective liquid biopsy‐based diagnosis and a potential prognosis method for colon cancer using cfDNA.

## 1 INTRODUCTION

5‐methylcytosine (5mC) is one of the most important epigenetic modification that impacts global gene expression in mammals. Genome‐wide 5mC pattern is extensively reshaped during cell differentiation and mammalian cell development, as well as the development and treatment of the human diseases such as cancer.^1^, ^2^ In contrast to methylation, demethylation has been observed in mammalian genome for decades^3^, ^4^, ^5^, ^6^ including passive demethylation and active demethylation. Active demethylation is mediated by the ten‐eleven translocation (TET) family of dioxygenases that oxidize the 5mC modification to 5‐hydroxymethylcytosine (5hmC),^7^, ^8^ and further to 5‐formylcytosine (5fC), and 5‐carboxylcytosine (5caC)^9^, ^10^, ^11^ in a step‐wise manner. 5‐Hydroxymethylcytosine is not only the ‘intermediate’ during active demethylation pathways but also acts as a stable DNA mark that plays crucial epigenetic roles.^12^, ^13^, ^14^, ^15^, ^16^, ^17^, ^18^ Recently developed genome‐wide sequencing methods of 5hmC in various mammalian cells and tissues associate the distribution as a marker for active gene expression.^19^, ^20^, ^21^, ^22^, ^23^, ^24^, ^25^ 5‐Hydroxymethylcytosine is enriched in enhancers, gene‐bodies and promoters, and the fold changes in 5hmC level in 5hmC sequencing maps correlate with changes in gene expression levels in RNA‐Seq.^25^, ^26^

Cell‐free DNA (cfDNA) originating from different tissues into the circulating blood has been studied for a long time and has shown significant roles in clinical diagnosis,^27^ boosting the rapid development of the liquid biopsy‐field. cfDNA‐based biomarkers and detection tools offer substantial advantages over the intrinsic methods. The minimally invasive blood test has revolutionary potentials in clinics, having higher patient compliance, is clinically convenient, cost‐efficient and enables dynamic monitoring.^28^ Tumour‐related somatic mutations in cfDNA have been well studied shown to be consistent with the mutations found in tumour tissue, which has been applied in dynamic monitoring of the drug treatment. However, mutation frequency is low and hard to provide the information on tissue of origin, which hampers the application of mutation detection methods into a universal diagnostic or prognostic method. Sensing hypermethylated 5mC region has been shown as an effect way to detect tumour biomarkers from plasma.^29^, ^30^, ^31^ 5‐Hydroxymethylcytosine could serve as a parallel or more valuable biomarker for human diseases because it represents active gene expression changes compared to the gene silencing effect in the hypermethylation region. Once the 5hmC patterns can be sensitively and robustly detected, disease‐specific biomarkers could be identified.

Next‐generation sequencing is an advanced platform for detecting cytosine modification patterns because of its ability in capturing complex information. A selective chemical labelling‐based technology platform named 5hmC‐Seal has been applied to map 5hmC using low‐input DNA. Here, we showed that 5hmC‐Seal technology is robust for 5hmC profiling in low‐input DNA including cell‐free DNA. We detected differentially expressed 5hmC regions in both tissue gDNA and cfDNA in colon cancer patients. This technology showed high potential in real world clinics.

## 2 METHODS

### 2.1 Study design and sample preparation

A total of five colorectal cancer patients and three precancerous adenoma patients above 20 years were diagnosed in Zhongshan Hospital at Fudan University, China from July 2017 to September 2017. All specimens were collected from patients who were newly diagnosed, and were about to undergo surgery, as well as received no neoadjuvant therapy pre‐operation. The control plasma samples were collected from healthy individuals who visited the clinic for medical examination. This study was approved by the Ethical Committee of Medical Research, Shanghai Zhongshan Hospital of Fudan University, and written informed consents were obtained from all patients before the surgery.

Paired cancer tissues and para‐carcinoma tissues from five patients were stored at –80°C after surgical removal. The gDNA was isolated using the Quick‐gDNA MicroPrep (Zymoresearch, California, USA) kits according to the manufacturer's protocol. Ten plasma samples were collected from colon cancer patients and healthy individuals. The cfDNA was isolated by the QIAamp Circulating Nucleic Acid Kit (Qiagen, Santa Clarita, CA) according to the manufacturer's protocol.

### 2.2 Spike‐in probe

In this study, two similar spike‐in probes with unique sequences named 5hmC spike‐in and no5hmC spike‐in were designed. 5hmC spike‐in: 5′‐CTGTCATGGTGACAAAGGCATCC*GGCAGAAATGCCCACACAGCCTCTT TAACCAGCACGCCAACCGCCTCTGCTTCGGCCCTGGTCACGCAGCTGACAAGGTCTTCATAATAGAGAAATCCTG‐3′, C*—5hmC modifications. no5hmC spike‐in: 5′‐CTGTCATGGTGACAAAGGCATCGCAGCGAAATGCCCACACAGCCTCTT TAACCAGCACGCCAACCGCCTCTGCTTCGGCCCTGGTCACGCAGCTGACAAGGTCTTCATAATAGAGAAATCCTG‐3′.

### 2.3 5hmC‐Seal‐seq library preparation and sequencing

The 5hmC library preparation and sequencing were conducted as described previously.^32^ Briefly, we applied the T4 bacteriophage β‐glucosyltransferase to transfer an engineered glucose moiety containing an azide group onto the hydroxyl group of 5hmC. Then, 5hmC‐containing DNA fragments were labelled by chemical modification with biotin on the azide group for further affinity enrichment. PCR amplification was utilized to amplify the captured DNA fragments, followed by the purification of the PCR products using AMPure XP beads according to the manufacturer's instructions. Afterwards, the sequencing was performed on the Illumina NextSeq 500 platform.

For robustness validation, a standard 5hmC‐containing gDNA (500 ng, Catalog# D5018, Zymo Research) isolated from human brain and spleen tissue was tested by 5hmC‐Seal approach. This set is an ideal control for detection and quantification methods against 5mC and 5hmC as both the modified cytosines are present at physiologically relevant levels and loci.

### 2.4 Sequencing data processing

Illumina reads were poste‐processed and mapped to the human hg19 assembly using the bowtie program with default parameters. We used samtools^33^ to generate bigwig files, and deepTools^34^ was adopted to plot the line chart of signal distribution. Model‐based analysis of ChIP‐seq (MACS)^35^ was used to identify the 5hmC‐enriched regions (peaks) in each sample (the qvalue cut‐off to call significant regions is 0.05). We used FeatureCount^36^ to determine feature counts on gene body for further study. Euclidean distance based on rlog‐transformed 5hmC signals was used to evaluate the difference between samples, the Bioconductor DESeq2 package^37^ was applied to detect the genotype‐specific genes, and r package ‘pheatmap’ (https://cran.r-project.org/web/packages/pheatmap/index.html)^38^ was used to visualize the distance in a heatmap figure. Gene Ontology (GO) (http://www.geneontology.org/) term analyses was performed by r package ‘clusterProfiler’ (http://bioconductor.org/packages/release/data/annotation/html/org.Hs.eg.db.html)^39^ and visualized as functional gene network by r package ‘pathview.’^40^

## 3 RESULTS

**Figure 1:**
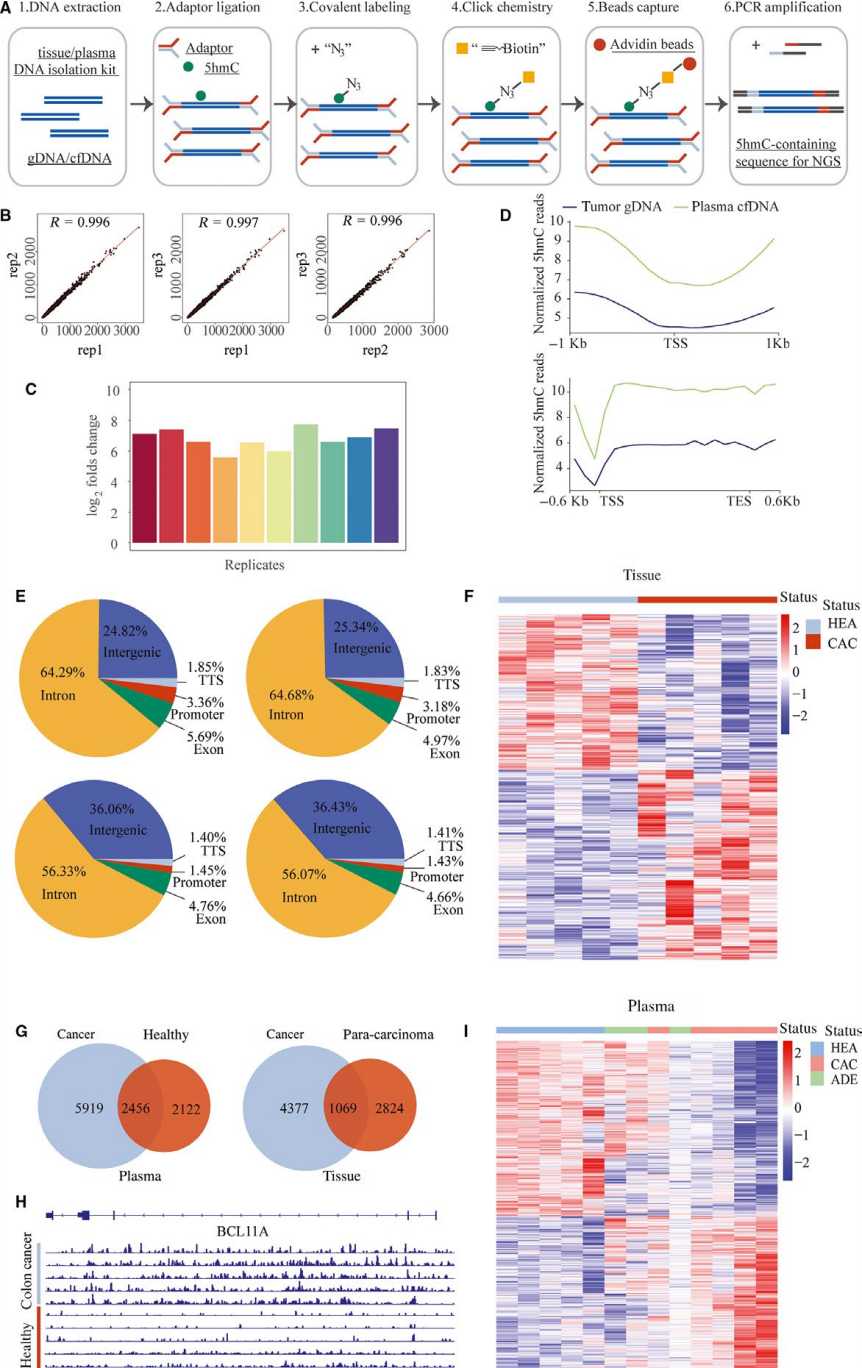
5‐Hydroxymethylcytosine (5hmC)‐Seal profiling in colorectal cancer samples. A, Workflow of 5hmC‐Seal profiling from gDNA and cell‐free DNA (cfDNA) is shown. Purified fragmented gDNA and cfDNA is ligated with sequencing adaptors. 5‐Hydroxymethylcytosine‐containing fragments are selectively labelled with a biotin group in a two‐step reaction. The biotin‐labelled fragments are captured on the streptavidin beads, followed by PCR amplification and next‐generation sequencing (NGS). B, Scatterplots showing correlation between 5hmC‐Seal replicates with Pearson correlation (r) displayed. From left to right: correlation between replicate 1 and replicate 2, between replicate 1 and replicate 3, between replicate 2 and replicate 3. C, Column diagram showed the log2 enrichment fold of 10 replicates. D, Normalized 5hmC reads from gDNA and cfDNA matched in TSS and gene body regions. E, Peak distribution of 5hmC in four groups of samples. Left to right and top to bottom, tumour tissue samples; para‐carcinoma tissue; plasma of tumor patients; plasma form healthy individuals. F, Heatmap shows clustering of gDNA samples from tumour tissue (CAC) and para‐carcinoma tissues (HEA). Genes and samples were clustered by Euclidean distance using centred rlog‐transformed expression counts. G, Venn diagram showing the overlap of differential 5hmC peaks in gDNA and cfDNA samples. H, Genome browser views of 5hmC signals detected in BCL11A gene region from libraries generated with gDNA from tissue colon cancer patients and para‐carcinoma tissues. I, The heat map shows clustering of cfDNA samples from plasma of colon cancer patients (CAC), precancerous adenoma (ADE) and healthy individuals (HEA). Genes and samples were clustered by Euclidean distance using centred rlog‐transformed expression counts

**Figure 2:**
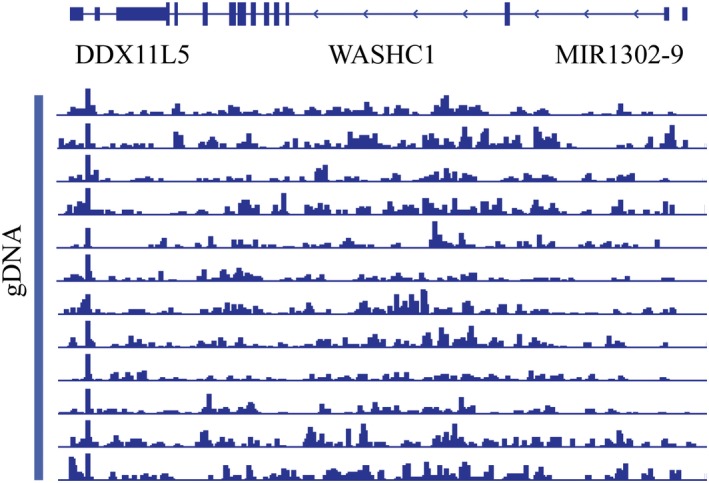
Representative genome browser views of 5‐hydroxymethylcytosine (5hmC) signals. Genome browser views of 5hmC signals detected in indicated region from libraries generated from standard 5hmC gDNA replicates showing reliable 5hmC signal distribution

First, we tested the robustness of 5hmC‐Seal approach. Although the core chemistry of this technology has been developed for years, a systematic study on the robustness of this technology as a clinical kit is still lacking. We used a commercially available standard 5hmC‐containing gDNA (Zymo research) isolated from human brain and spleen tissue to ensure the consistency of the sample. The workflow of 5hmC‐Seal profiling method in this study was shown in Figure 1A. As published before, the labelling, capture and washing steps were further optimized for capturing 5hmC‐containing DNA fragments from low‐input DNA.^32^, ^41^ To verify the reproducibility of the technology, we repeated the library construction step of a 10‐ng standard DNA sample for 10 times continuously for 10 days with three different technicians, and the correlation between the 10 replicates was analysed via Pearson correlation. To our delight, the 10 5hmC profiling maps were highly correlated to each other (*R* > 0.99, three representative correlation analysis results shown in Figure 1B). This test was expanded to three independent batches of reagents with a continuous 20‐day test and the similar high quality (data not shown) was maintained. To further study the reliability of the global 5hmC pattern, we visualized the 5hmC signals on the genome browser view, as shown in Figure 2; the 5hmC signals were distributed consistently on the genome among different replicates. Next, we designed two spike‐in probes with only limited differences near the oligo ends to function as different sequences during analysis, one contains the 5hmC modification in the middle and one contains normal cytosine. With this design, the sequence content between the two spike‐ins is almost identical, thus this could be the ideal model for testing the 5hmC capture efficiency during the 5hmC‐Seal assay.

Next, we applied this method in paired colorectal cancer samples, pairs of carcinoma tissues and adjacent tissues from five colorectal cancer patients, as well as plasma samples from colorectal cancer patients (n = 5) and health controls (n = 5). In general, the average all reads in 20 samples was 30 million bp. The all reads in the five cancer plasma samples were higher, which ranged from 34 million to 47 million bp with a mean value of 41 million bp. We revealed that 5hmC was enriched within the gene body. In addition, the genomic enrichment pattern of 5hmC was consistently observed in both tissue gDNA and blood cfDNA (as shown in Figure 1D). Interestingly, the gene body enrichment of the blood cfDNA is much higher than the enrichment pattern from tissue gDNA, indicating an increased 5hmC level in the gene body of cfDNA compared to the tissue. Furthermore, we calculated the distribution of 5hmC sequencing peaks in various genetic region categories including intergenic, intron, exon (5′untranslated region (UTR), 3′UTR, coding‐exons), Transcriptional start site (TSS) and promoter (−2 kb to +0.5 kb relative to TSS) regions to analyse the 5hmC distribution along the genomic features. 5‐Hydroxymethylcytosine peaks were enriched within the gene body and kept similar distribution patterns between tumour samples and healthy controls, no matter in gDNA or cfDNA (Figure 1E). We observed different 5hmC peak distribution in the intron and intergenic region between cfDNA sample and tissue sample. Higher peak percentage has been found in intron in tissue samples, while blood samples have higher percentage in intergenic regions.

**Figure 3:**
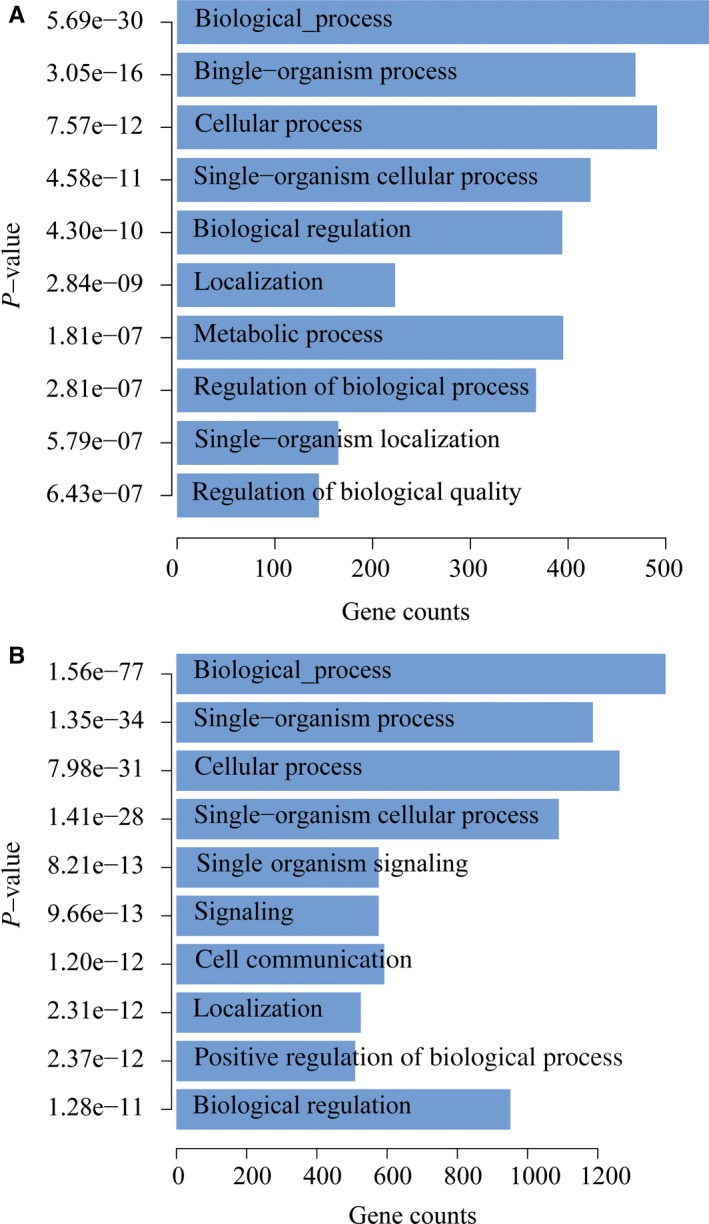
Gene Ontology analysis shows the top categories of 5‐hydroxymethylcytosine (5hmC) signal differential genes. A, Top categories of 5hmC signal differential genes identified from cancer tissues and para‐carcinoma tissues. B, Top categories of 5hmC signal differential genes identified from plasma of cancer patients and plasma of healthy person

To address the technology potentials in clinical diagnosis and prognosis, we tested if there are specific 5hmC profile patterns between the tumour samples and para‐carcinoma tissues. As shown above, the 5hmC reads were mainly distributed in the gene body, therefore, define that the differential 5hmC region on the gene body could be a robust method to cost‐effectively collect enough reads in statistics, which is important for a practical clinical purpose. We calculated and normalized the 5hmC reads for each gene body from each sample. Differential genes with remarkable 5hmC level difference were identified and listed in Table S1. Then, we applied an unsupervised hierarchical clustering of those differentially modified 5hmC loci, and the samples from colorectal cancer patients were distinctly separated from healthy individuals (Figure 1F). We used Venn diagram to show the overlap of differential 5hmC peaks between cancer samples and healthy control samples. To gain insight into the dynamics of the 5hmC changes, we quantified the number of peaks that were gained or lost in each group. Comparison of plasma from cancer patients and healthy individuals revealed a substantially higher number of peaks gained in cancer (5919) than that which is lost in cancer (2122). Similarly, comparison between cancer tissue and para‐carcinoma tissues indicated a higher number of peaks gained in the cancer tissue (4377) than lost in the cancer tissue (2824). The overlap peaks between cancer plasma and healthy plasma was 2456, and 1069 peaks coexist in cancer tissues and para‐carcinoma tissues (Figure 1G). When we visualized the 5hmC signal in loci, the 5hmC peaks are strongly increased in the cancer tissue across the gene body of the representative genes (Figures 1H & 2), indicating that this 5hmC signal change could be a long‐distance event. Gene Ontology enrichment analysis indicated that these 5hmC‐change‐related genes may be associated with important biological pathways during cancer development (as shown in Figure 3A).

Finally, we tested if this methodology could generate the 5hmC differential genes among cancer patients, healthy controls and pre‐cancerous adenoma patients from plasma, which is the key to evaluate the potential methods in non‐invasive clinic diagnosis. We compared five plasma samples from colon cancer patients, three plasma samples from adenoma patients and five plasma samples from healthy individuals. We first identified the differential 5hmC genes (*P* < 0.05) and unsupervised hierarchical clustering with these genes was applied to all the samples. As shown in Figure 1I, the differential 5hmC genes could clearly cluster the samples from cancer patients and healthy individuals into two classes, meanwhile, the three adenoma samples hold intermediate 5hmC signal patterns between the healthy and colorectal cancer (CRC) individuals. The results indicate the potential application of 5hmC change signals in colon cancer early, even in the pre‐cancerous adenoma. To better understand the differential genes found in the blood, GO analysis was adopted, the results revealed that the differential 5hmC genes from blood samples exhibited similar pathways with that from tissue samples (Figure 3B).

## 4 DISCUSSION

In this study, spike‐in probes with only limited difference were adopted to verify the reliability of the 5hmC‐Seal method for 5hmC profiling. As a result, the sequence content between the two spike‐ins is almost identical, thus this could be the ideal model for testing the 5hmC capture efficiency during the 5hmC‐Seal assay. The average log2 fold enrichment of the spike‐in probes in 10 replicates was around seven (Figure 1C), which is consistent with the published data,^21^, ^32^ indicating the high 5hmC capture affinity and the robustness of 5hmC‐Seal profiling technology in such low‐input DNA.

Next, we applied this method in pairs of carcinoma tissues and adjacent tissues from five colorectal cancer patients, as well as plasma samples from colorectal cancer patients and health controls. We revealed that 5hmC was enriched within the gene body, while it was under‐represented in regions near TSS, which is consistent with the previous studies.^23^, ^32^

**Figure 4:**
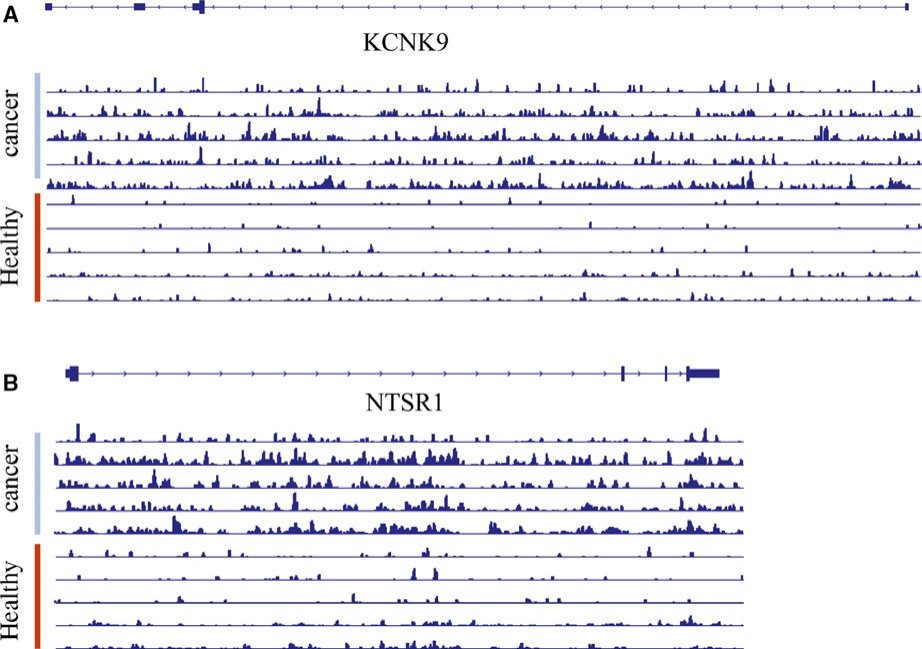
Representative genome browser views of 5‐hydroxymethylcytosine (5hmC) signals from cancer tissues and para‐carcinoma tissues. Genome browser views of 5hmC signals detected in TWIK‐related acid‐sensitive K+ channel 3 (KCNK9) (A) and Neurotensin Receptor 1 Gene (NTSR1). B, gene regions of libraries generated from gDNA of cancer tissues (cancer) and para‐carcinoma (healthy) tissues, showing reliable 5hmC signal distribution

Decreased global 5hmC levels in various cancer tissues were reported in previous studies,^15^, ^42^ however, 5hmC gained peaks have been discovered as well.^32^, ^43^ Our data again indicated that the decreasing global 5hmC level may not represent the 5hmC change in genome in diseases, but rather a total level change in background. When we visualized the 5hmC signal in loci, the 5hmC peaks are strongly increased in the cancer tissue across the gene body of the representative genes (Figures 1H & 4), indicating this 5hmC signal change could be a long‐distance event.

In summary, we established a continuous quality control assay for testing the repeatability and stability of 5hmC‐Seal technology, and further proved that this 5hmC profiling method is robust in mapping low‐input DNA in a cost‐effective manner. 5‐Hydroxymethylcytosine occurs in gene bodies, indicating that the genomic locations of 5hmC is associated with actively expressed genes. Utilizing a robust and highly efficient profiling‐based approach to map 5hmC samples from patients with cancer, we were able to identify differential 5hmC peaks and genes that can distinguish tumour tissues from the adjacent normal tissues. Our study showed that we can also identify differential 5hmC signals in plasma from colon cancer patients and healthy controls. On account of CRC are mostly sporadic and develop from removable precancerous lesions (adenomas) and curable early stage cancer, thus screening for CRC has high potential could reduce morbidity and mortality.^44^ This study provides the potential value and feasibility of 5hmC‐Seal approach on CRC early detection. Moreover, the results indicated that 5hmC profiling of cfDNA from liquid biopsies could serve as parallel or more valuable markers for non‐invasive diagnosis and prognosis of various diseases.

## 5 CONCLUSIONS

We demonstrated the robustness of the 5hmC‐Seal method in tissue and cfDNA as potential biomarkers. Moreover, this study provides the potential value and feasibility of 5hmC‐Seal approach on CRC early detection. We believe this strategy could be an effective liquid biopsy‐based diagnosis and potentially serve as a prognosis method for colon cancer using cfDNA.

## ETHICS APPROVAL AND CONSENT TO PARTICIPATE

This study was approved by the Ethical Committee of Medical Research, Shanghai Zhongshan Hospital of Fudan University, and written informed consents were obtained from all patients before the surgery.

## CONSENT FOR PUBLICATION

Not applicable.

## AVAILABILITY OF DATA AND MATERIAL

The datasets used and/or analysed during the current study are available from the corresponding author on reasonable request.

## CONFLICT OF INTEREST

The authors declare that they have no competing interests.

## AUTHORS' CONTRIBUTIONS

PT Gao, XY Lu, SL Lin, MY Cai, YH Cui, PH Zhou designed the research and collected the samples from patients. PT Gao, Y Zhu, SL Lin and YQ Song and performed the experiments. Y Sui, J Lin, XYJ Liu and XY Lu analysed the data. PT Gao, YH Cui, XY Lu, YS Zhong and PH Zhou were the major contributors in writing the manuscript. All authors read and approved the final manuscript.
